# Supplementary material for: CD4+ T Cell Fate Decisions Are Stochastic, Precede Cell Division, Depend on GITR Co-Stimulation, and Are Associated With Uropodium Development
Source: Front Immunol. 2018 Jun 18;9:1381. doi: 10.3389/fimmu.2018.01381 (PMC6015874; doi:10.3389/fimmu.2018.01381)
Supplement: Supplementary file 2 [file data_sheet_1.PDF]

Supplementary Figure 1: Additional analysis of data from Figure 5

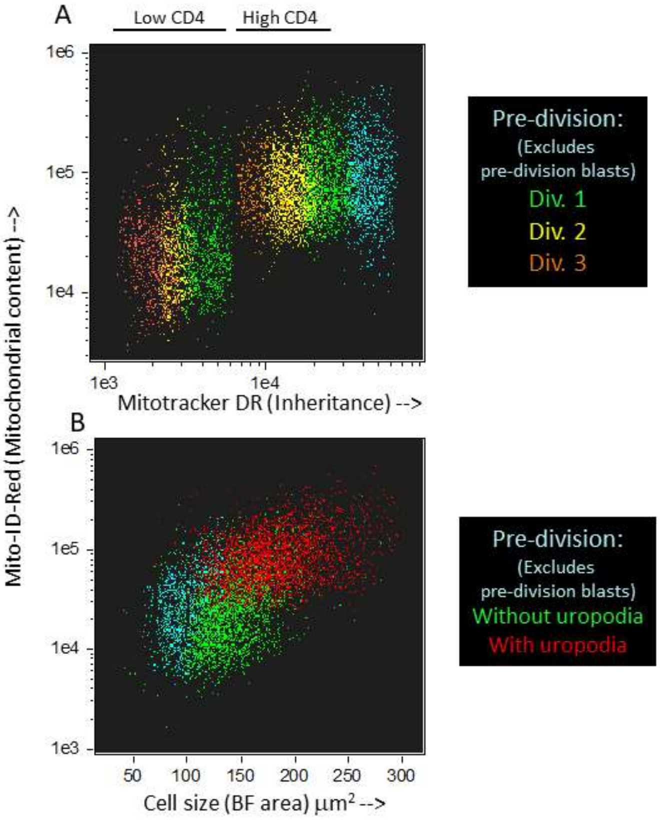

A. The data of Figure 5 is presented in an alternative format to further clarify the independent behaviour of the two populations with either high (Mito-ID-Red high) or low (Mito-ID-Red low) numbers of mitochondria as they proliferate. All images were gated for focus and live (low BF contrast) and singlet (BF area < 200µm, BF aspect ratio > 0.7) cells. Each cell image represented on the scatter plot is colour coded according to the number of divisions completed by CTV dilution. Cells with undiluted CTV (pre-division, blue dots) excluded blasts (BF area >90µm) as these were transitioning between the high and low states and, if included, overlapped and obscured the populations shown. Each subsequent CTV dilution was then tightly gated on the distinct Mitotracker DR and CD4 high versus low populations within each CTV division peak and all the gated populations combined shown as green, yellow or orange coloured dots (representing 1, 2 or 3 divisions, respectively). This way of showing the data emphasises the major loss (approx. 1 log or 90%) of inherited Mitotracker DR staining that occurs entirely before completion of the first cell division. This generates a separate and consistently low Mito-ID-Red (total mitochondrial content) and CD4low population distinct from the constantly high Mito-ID-Red staining and regular binary inheritance of Mitotracker DR staining in the CD4 high cells.

B. The same data is presented showing the relationship between cell size, mitochondrial content and the presence or absence of uropodia. Blue dots are pre-division cells excluding blasts, red and green dots are cells with and without uropodia, respectively, combined for divisions 1-3, showing that cells with uropodia and high mitochondria do also tend to be larger (171μm^2 compared to 137μm^2 without uropodia and 96μm^2 for pre-division cells excluding blasts).

Supplementary Figure 2: Proliferating high and low mitochondrial staining CD4+ T cell populations have a similar mitochondrial DNA content

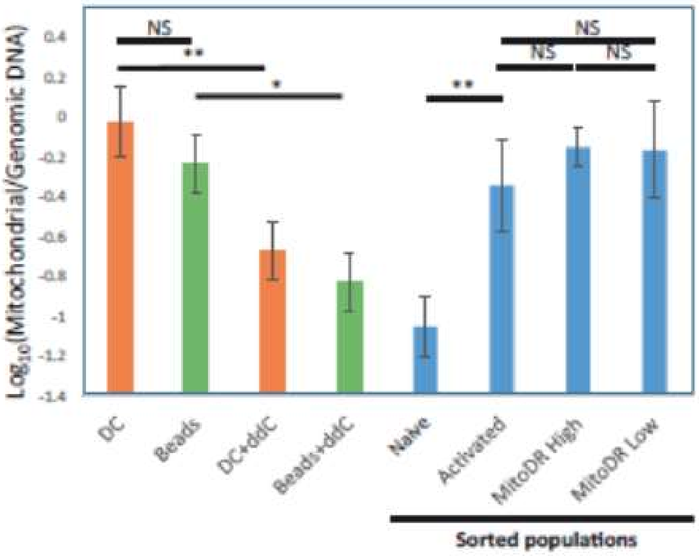

CTV-labelled A1RAG CD4+ T cells were stimulated for 3 days with either bmDC+Dby peptide or CD3/CD28 beads plus IL2 and TGFβ as in previous experiments. Some cultures included di-deoxy cytidine (ddC), which inhibits mitochondrial DNA replication, such that it is diluted with each cell division, eventually generating cells with normal numbers of non-functional mitochondria lacking any genome (1). On day 3, bmDC stimulated cultures were labelled with Mitotracker DR and were sorted for cells which had diluted CTV (divided once or more) and into MitoDR high and low staining. Cells which had not diluted CTV were also sorted into high (activated) and low (naïve) MitoDR staining populations and PCR performed to determine the ratio of a mitochondrial gene (Cyclooxidase1) to a nuclear (genomic) gene (NDUFV1) (2). Error bars are geometric SDs, P values (ANOVA, n=6 replicates across 2 experiments) NS = P >0.05; * P<0.05; ** P<0.01.

We also attempted to sort the MitoDR high and low populations, on day 2 or 3 of culture, for further studies into their functional properties. We found, however, that re-stimulation was then required for both proliferation and survival, and that under these conditions neither population maintained any uropodia nor did they keep the levels of mitochondria they had pre-sorting. This suggests that their cell fates may also be sensitive to the environment during sorting and handling and would probably confound any interpretation of additional functional experiments.

1. Nelson I, Hanna MG, Wood NW, Harding AE. Depletion of mitochondrial DNA by ddC in untransformed human cell lines. *Somat Cell Mol Genet* (1997) 23(4):287-90. Epub 1997/07/01. PubMed PMID: 9542530.
2. Guo W, Jiang L, Bhasin S, Khan SM, Swerdlow RH. DNA extraction procedures meaningfully influence qPCR-based mtDNA copy number determination. *Mitochondrion* (2009) 9(4):261-5. doi: 10.1016/j.mito.2009.03.003. PubMed PMID: 19324101; PubMed Central PMCID: PMC2798162.

Supplementary Figure 3: Common artefacts that misleadingly suggest asymmetric cell divisions

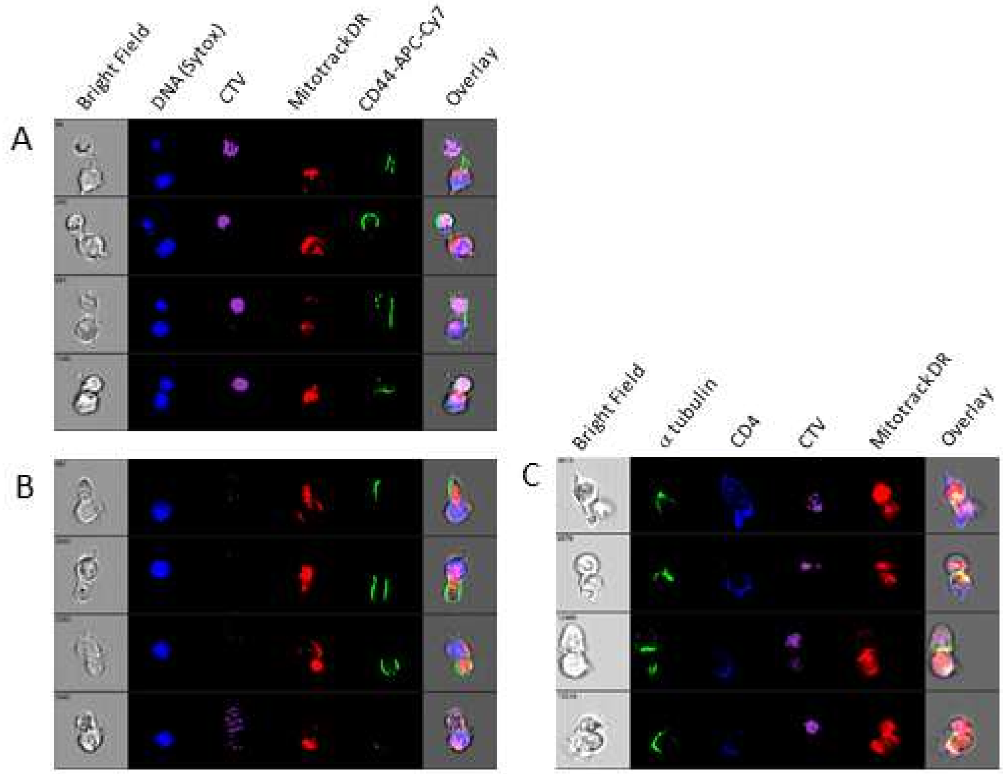

The images shown are examples taken from the very same data set as that shown in Figure 6. These images were identified by first gating for focussed, live, CTV and CD4 positive T cells in G_2/M by DNA content, low DNA aspect ratio (<0.8) and low BF aspect ratio (<0.8). Mitotic candidates were then selected by visual inspection of the BF images for those with an apparent cytoplasmic bridge. Panel A shows the most common reason for observing a false asymmetry due to incorrectly identifying cell conjugates/doublets as mitotic cells in cytokinesis. These cannot be true mitotic cells as the two “daughters” come from different cell divisions, indicated in the examples shown by only one cell in the conjugates containing a high level of CTV. A more detailed analysis (not shown) indicated that such cell conjugates could be found in random combinations between cells with CTV staining from all cell generations, were more frequent in samples with uropodia present, and were at least 10x more frequent than true mitotic cells across many different samples analysed. In other experiments (panel C) these conjugates between cells from different CTV generations could even contain apparent tubulin bridges, which might suggest the conjugated cells were forming some form of “synapse”. Panel B shows a different set of images that were mistakenly selected as mitotic candidates on a similar basis as in Panel A. In panel B, however, one of the “daughter” cells in each image can be seen to contain no DNA (while the other has the DNA content of a cell in G_2) and stains strongly for CD44. These are simply single cells in G_2 but not in mitosis nor cytokinesis, with large $CD44^+$ uropodia mimicking a daughter cell.

Supplementary Figure 4: Apparent asymmetric cell divisions due to distortion during fixation

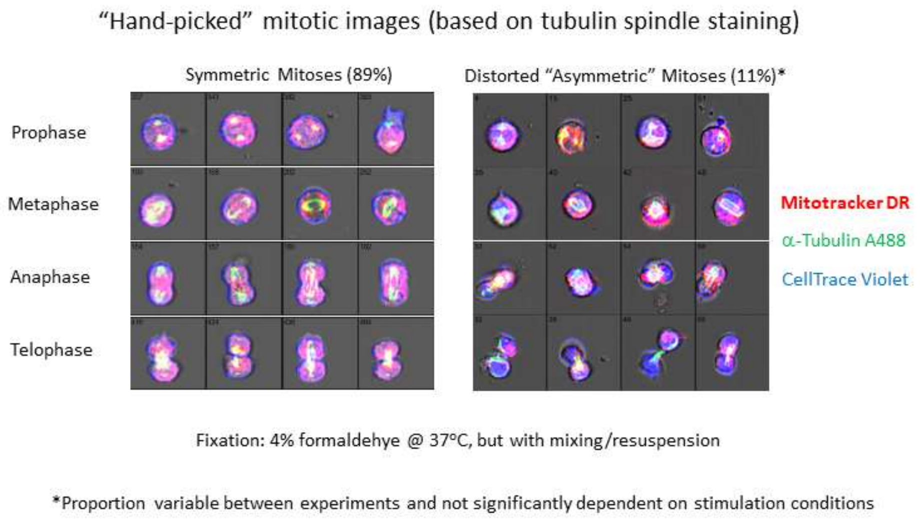

Example images are shown of cells at various stages of mitosis. These were visually selected as described in Supplementary Figure 3, but from a different experiment, and using the additional staining with alpha and gamma tubulin antibodies to stain the mitotic spindle for determining the specific phases of mitosis. Cells were fixed with 4% formaldehyde in PBS at 37°C for 15 mins, but after resuspension rather than without disturbing them. Mitoses that appeared to be asymmetric for mitochondrial staining were generally distorted, but still only comprised a minority of all mitotic cells (11% in the example shown, although this was highly variable in different experiments) and were found in similarly processed samples regardless of whether they were stimulated with DC+peptide, DC+peptide+rapamycin, or CD3/CD28 beads. These distortions were frequently found after cell harvesting/resuspension, centrifugation, or room temperature fixation, but could be minimised by performing all steps up to and including fixation at 37°C in situ, without disturbing the cells. Note that we found that activated, primary $CD4^+$ T cells were particularly sensitive to loss or distortion of cells in mitosis during manipulation, lower temperatures or incomplete handling media when compared to a T cell line (EL4) in which we could readily observe a high frequency of symmetric telophases under most conditions.
